# Changes of operative performance of pulse pressure variation as a predictor of fluid responsiveness in endotoxin shock

**DOI:** 10.1038/s41598-022-06488-x

**Published:** 2022-02-16

**Authors:** Jorge Iván Alvarado Sánchez, Juan Daniel Caicedo Ruiz, Juan Jose Diaztagle Fernández, Gustavo Adolfo Ospina Tascon, Manuel Ignacio Monge Garcia, Guillermo Arturo Ruiz Narvaez, Luis Eduardo Cruz Martínez

**Affiliations:** 1 Department of Physiological Sciences, Faculty of Medicine, Universidad Nacional de Colombia, Bogotá, Colombia; 2 Department of Anaesthesiology, Centro Policlínico del Olaya, Bogotá, Colombia; 3 Department of Internal Medicine, Hospital de San José, Fundación Universitaria de Ciencias de la Salud, Bogotá, Colombia; 4 Department of Intensive Care, Fundación Valle del Lili, Universidad ICESI, Cali, Colombia; 5 Critical Care Unit, Hospital Universitario de Jerez de la Frontera, Jerez de la Frontera, Cádiz, Spain; 6 Instituto de Simulación Médica (INSIMED), Bogotá, Colombia

**Keywords:** Physiology, Experimental models of disease, Translational research

## Abstract

Several limitations regarding pulse pressure variation (PPV) use have been reported. Our aim was to describe changes in the PPV operative performance as a predictor of fluid responsiveness during the development of a swine endotoxin shock model and to assess hemodynamic variables associated with PPV changes. A swine porcine endotoxin shock model was established (*Escherichia Coli* 055:B5 endotoxin) in 7 pigs, and 3 pigs were included in the control group. The endotoxin was infused until the mean arterial pressure (MAP) dropped below 50 mmHg (TH0); then, the model animal was reanimated with fluids and vasopressors. We performed fluid challenges every hour for 6 h. ROC curve analysis and a linear mixed model were performed. The area under the curve of PPV decreased from 0.95 (0.81–1.00) to 0.60 (0.17–1.00) at TH0. Its cutoff increased from 10.5 to 22.00% at TH0. PPV showed an inverse relationship with stroke volume, mean systemic filling pressure, MAP, and systemic vascular resistance (SVR) (*p* < 0.001, AIC = 111.85). The PPV operative performance as a predictor of fluid responsiveness decreased with the progression of shock. This could lead to an inverse association between PPV and the following variables: MAP and SVR.

## Introduction

Appropriate fluid therapy is key for the management of critically ill patients in intensive care units or the operating room^1,2^. The use of intravenous fluid therapy based on physiological variables related to fluid responsiveness has been beneficial in these patients^3,4^, whereas its use without defined hemodynamic goals has been associated with an increased medical complication rate^5^.

Dynamic indices related to cardiac preload have been generally used as predictors of fluid responsiveness. A positive response to fluid challenge is usually defined as a 10–15% increase in cardiac output (CO) or stroke volume (SV)^6^. Among these indices, pulse pressure variation (PPV) has been used as a predictor of fluid responsiveness in mechanically ventilated patients in several clinical settings. However, some limitations have been described^7,8^.

Although this hemodynamic variable has been studied for 20 years, little is known about its operative performance as a predictor of fluid responsiveness during hypotensive states, such as septic shock. Moreover, Monge et al. showed that PPV is related to effective arterial elastance^9^, a variable that summarizes the features of arterial vascular load in humans^10^. Therefore, its operative performance and its cutoff as a predictor of fluid responsiveness could change in hypotensive states.

This study aimed to describe changes in the PPV operative performance and cutoff of PPV as a predictor of fluid responsiveness during the induction of endotoxic shock in a swine model. We also used a statistical model to explore the possible relationship between PPV and other hemodynamic variables that could explicate this decrease in the PPV operative performance.

## Materials and methods

### Protocol

The study was carried out in accordance with the principles for the care and use of animals in research established by the Guide for the Care and Use of Laboratory Animals (NIH Guide)^11^, Resolution 008430 of 1993 issued by the Colombian Ministry of Health, and Law 84 of 1989 issued by the Congress of Colombia, the “Estatuto Nacional de Protección de Animales” (National Statute for the Protection of Animals). Additionally, this research was conducted according to the Animal Research: Reporting of In Vivo Experiments (ARRIVE) guidelines^12^.

### Preparation of animals

This study was approved by the Bioethics Committee of the Faculty of Veterinary Medicine of Universidad Nacional de Colombia (CB-FMVZ-UN-031-19). The study was conducted at the simulation laboratory of Instituto de Simulacion Medica (INSIMED) from January to December 2019. Ten female pigs (Yorkshire 40 ± 1.0 kg; ± 4 months old) were premedicated using tiletamine-zolazepam (ZOLETIL, Virbac Colombia) at 4.4 mg/kg doses through intramuscular injection. Then, each pig was cannulated through the marginal ear vein, and anesthesia was induced through the inhalation of isoflurane at a 1.5 minimum alveolar concentration (MAC) using an anesthesia mask. Once under general anesthesia, the pig was intubated and mechanically ventilated with a tidal volume of 10 ml/kg and a respiratory rate of 20 breaths/minute. For the maintenance of general anesthesia, isoflurane at doses of 1.5 MAC was used. Additionally, in all animals, the internal jugular vein and the femoral artery were cannulated using a central venous catheter (ARROW CV-17702) and an artery catheter (PiCCO PV2015L20-A). The quality of blood pressure signals was tested using a rapid washout test. All measurements were performed with the animal in a supine position, considering the phlebostatic axis as the zero reference.

Animals were placed on a stationary operating table and thermoregulated using medical blanket warmers, keeping their body temperature at a minimum level of 38 °C. During the development of the model, intravenous fluid was administered using normal saline solution (SS) at an infusion rate of 3 ml/kg/hour.

### Measurements

A monitor with the pulse contour cardiac output system (TFT Mindray BeneVision N22 Patient Monitor) was used. CO was calculated as the average of three thermodilution boluses (20 ml of < 8 °C SS through the jugular venous catheter). SV was calculated as CO/heart rate (HR). Systemic vascular resistance (SVR) and PPV were automatically calculated by the PiCCO system. Finally, MSFP was estimated using the method of Parkin^13^: MSFP = 0.96 (CVP) + 0.04 (MAP) + 0.5 (CO).

### Experimental protocol

After completion of the surgical procedures, animals were allowed to stabilize MAP (variation < 10%) at least for 10 min. After hemodynamic stabilization, animals were assigned to the control group (3 animals) or the endotoxin group (7 animals).

Animals in the endotoxin group received a continuous infusion of endotoxin (LPS *E. Coli* 055: B5, Sigma, St. Louis, MO) through the central venous catheter at an infusion rate of 7 μg/kg/h that was increased every 10 min (7, 14, and 20 μg/kg/h) until reaching 20 μg/kg/h ^14^. The infusion of endotoxin ended when an MAP < 50 mm Hg for at least 10 min was achieved. Pigs in the control group were not administered endotoxin.

When an MAP < 50 mm Hg was reached in the endotoxin group a fluid load was administered at a dose of 20 ml/kg for 20–30 min through the central venous catheter. In the control group, a similar fluid load was administered at 3 h of observation. Then, a noradrenaline infusion at a dose of 0.05 mcg/kg/min was started after the fluid load, and the infusion rate was increased 0.05 mcg/kg/min every 5 min until an MAP of 65 mmHg was reached. Noradrenaline was not administered in the control group. After completing the protocol, all animals were euthanized by a certified veterinarian with a bolus of pentobarbital+diphenylhydantoin at 100 mg/kg (EUTHANEX), in accordance with the international criteria for this procedure established by the American Veterinary Medical Association (AVMA).

### Fluid challenges and times of measurements

Time zero (T0) was defined in the endotoxin group and the control group as the time immediately after hemodynamic stabilization. TH0 was defined in the endotoxin group as the time when an MAP < 50 mm Hg was reached. In the control group, this time was set at 3 h of observation. This time was selected in the control group because it was the median time required to reach an MAP < 50 mm Hg for at least 10 min during the pre-experimental standardization phase of the endotoxin models.

A total of 6 fluid challenges were performed in each group. The first three fluid challenges were performed every hour starting at T0 (T0, T1, and T2). Afterward, three fluid challenges were performed every hour starting at TH0 (TH0, TH1, and TH2). Hemodynamic measurements were performed each time. This approach allowed us to assure that both groups had the same amount of fluid administered and that the variables could be compared.

**Figure 1:**
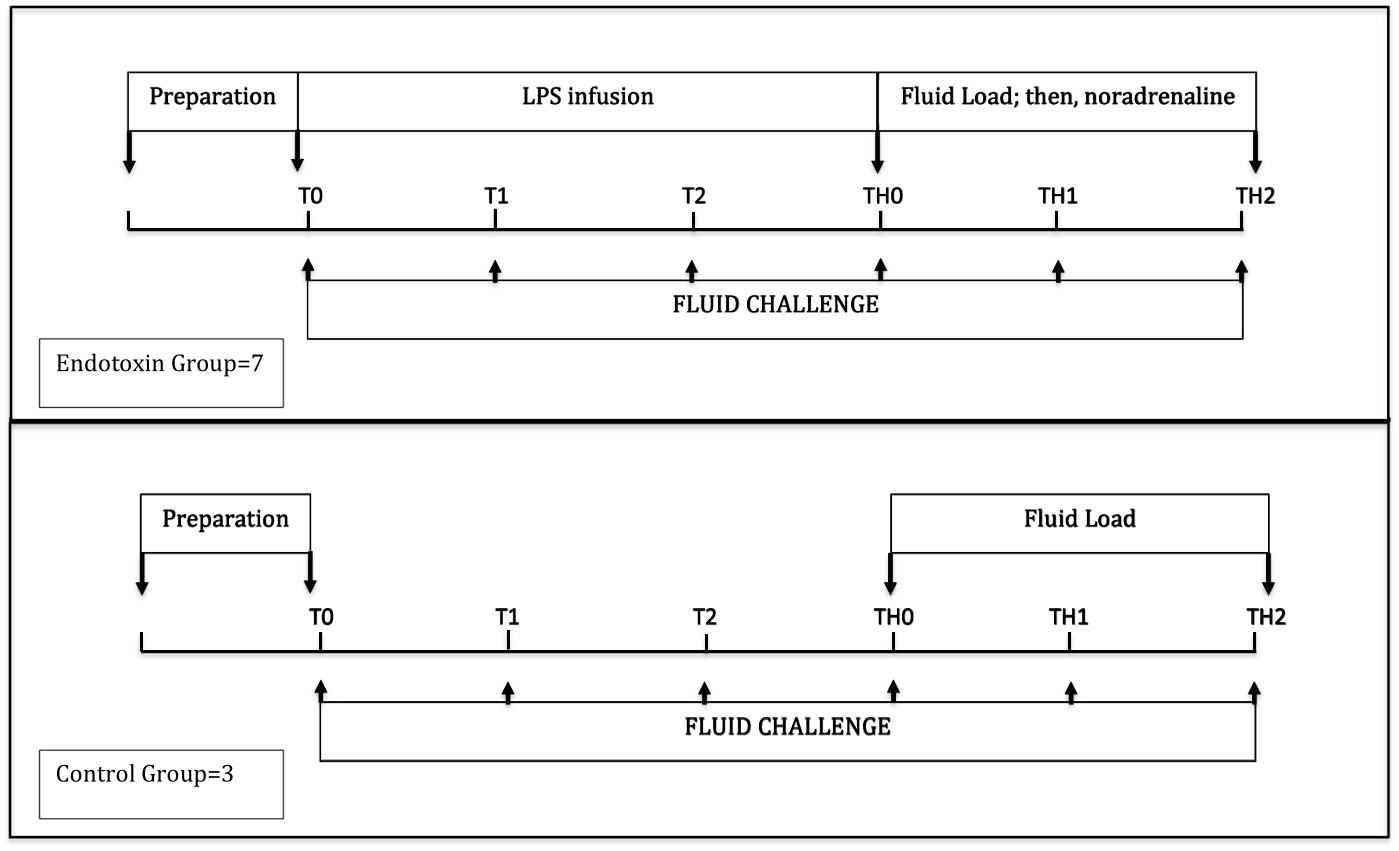
Study design.

All fluid challenges consisted of 4 ml/kg SS IV infused for 5 min through a central venous catheter. This is a standardized approach to perform a fluid challenge in humans^15^. Animals in which a fluid challenge induced an increase in CO > 10% were defined as fluid responders^6 6^. CO was measured by transpulmonary thermodilution in all cases. The study protocol is depicted in Fig. 1.

### Statistical analysis

Data are presented using medians and interquartile ranges. Receiver operating characteristic (ROC) curve analysis was conducted for each fluid challenge to assess changes in the PPV operative performance over time in the endotoxin group. Its 95% confidence intervals (95% CI) were calculated by the bootstrap method. The cutoff was calculated by maximizing the sum of sensitivity and specificity^16^.

Two-way analysis of variance (ANOVA) was conducted to assess differences between both groups. Additionally, one-way ANOVA was performed to determine changes in the variables over time in the endotoxin group. Post hoc analysis was carried out using the Bonferroni correction for multiple comparisons. Categorical variables were evaluated by the Chi-square test in each fluid challenge.

A linear mixed model was performed to determine the associations between PPV and SVR, MAP, SV, baseline PPV, MSFP, and the endotoxin or the control group. Time was used as the random effect, the variables in the model were assessed by the restricted maximum likelihood (REML), and the contribution on each variable was quantified using the estimated value and its standard deviation. Models were compared using the Akaike information criterion (AIC), the Bayesian information criterion (BIC), and the REML. The models with the lowest AIC and BIC were considered the best models.

Data were analyzed using R statistical software^17,18^. A *p* value < 0.05 was considered statistically significant for all analyses.

## Results

**Figure 2:**
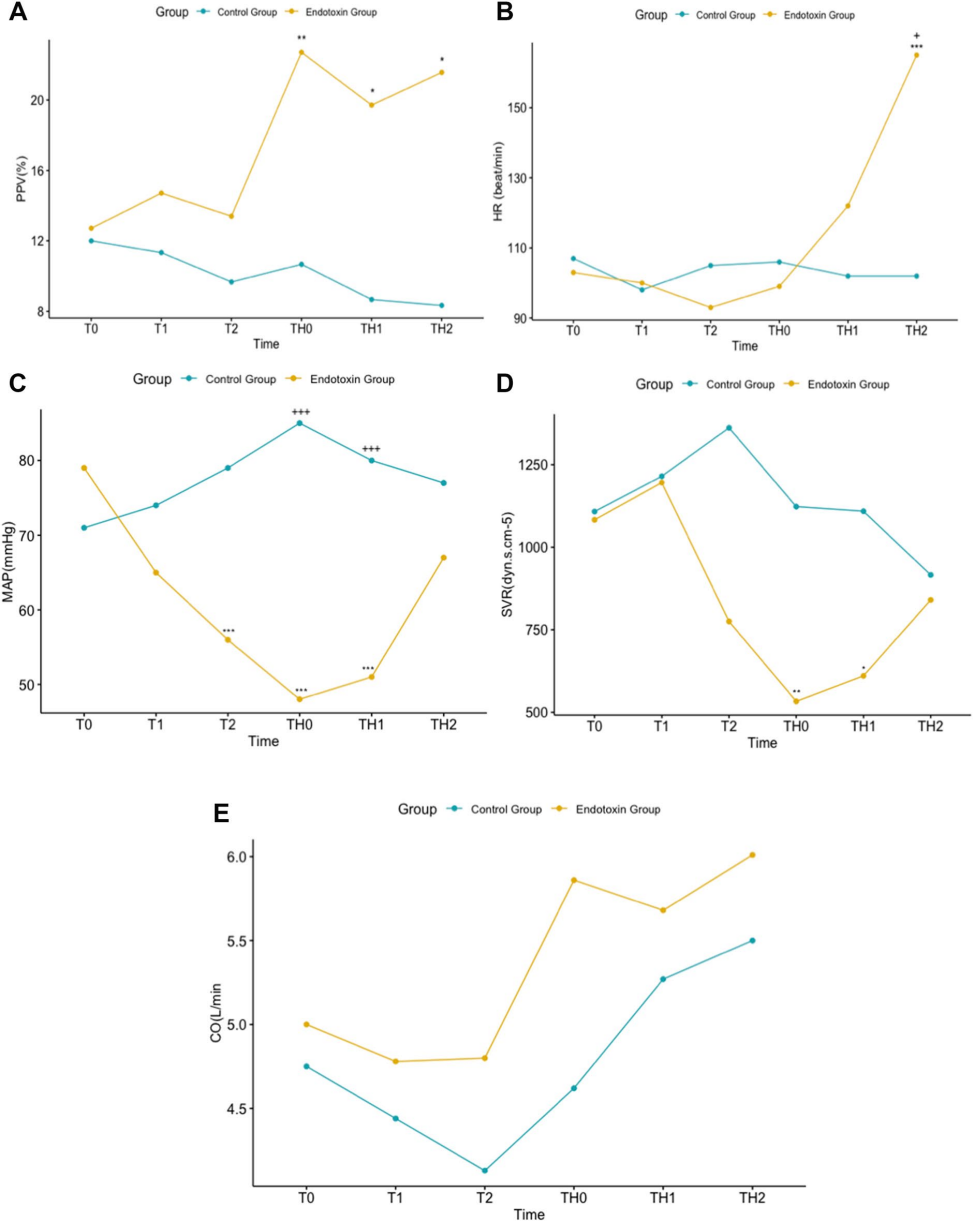
Changes of hemodynamic variables for 6 h of observation. (**A**) changes of pulse pressure variation (PPV); (**B**) changes of heart rate (HR); (**C**) changes of mean arterial pressure (MAP); (**D**) changes of systemic vascular resistance (SVR); (**E**) changes of cardiac output (CO). += *p* < 0.05, ++ = *p* < 0.01, +++ = *p* < 0.001 to comparison between Control and Endotoxin Group. *= *p* < 0.05, **= *p* < 0.01, ***= *p* < 0.001 to comparison between each time and T0 time on Endotoxin group.

An MAP < 50 mmHg was reached between 2 and 3 h after the start of endotoxin infusion. The changes in the hemodynamic variables are shown in Table 1 and Fig. 2. Systolic arterial pressure (SAP), diastolic arterial pressure (DAP), and MAP decreased, and their levels were lower in the endotoxin group. From T2 to TH1, SAP, DAP, and MAP were lower in the endotoxin group than in the control group (*p* < 0.05) (Table 1 and Fig. 2). Additionally, in the endotoxin group, these variables decreased over time (*p* < 0.05). SVR showed similar behavior when measured at TH0 and TH1 (*p* < 0.05) (Fig. 2D). The changes in the remaining variables are shown in Table 1.

**Table 1:** Hemodynamic variables.

Variable	T0	T1	T2	TH0	TH1	TH2
**HR (beat/min)**						
Control group	109.00 (106.00–110.00)	98.00 (94.50–107.50)	105.00 (88.00–113.50)α	106.00 (97.00–114.00)	102.00 (95.00–112.00)	102.00 (90.50–115.00)+
Intervention group	95.00 (91.00–105.50)	100.00 (98.50–106.50)	93.50 (82.00–94.00)	99.00 (94.50–117.50)	122.00 (98.50–138.00)	165.00 (131.00–187.00)***
**SAP (mmHg)**						
Control group	103.00 (92.50–104.50)	97.00 (95.00–101.00)	99.00 (97.00–99.50)+	105.00 (95.50–107.00)+ ++	100.00 (96.00–109.50)+ +	97.00 (96.50–100.50)
Intervention group	105.00 (89.00–111.50)	96.00 (89.50–115.50)	82.00 (76.00–92.00)**	68.00 (62.50–76.00)***	77.00 (71.50–79.00)***	95.00 (93.50–101.00)
**DAP (mmHg)**						
Control group	68.00 (60.00–69.00)	64.00 (61.50–69.00)	69.00 (64.50–71.50) +	75.00 (63.00–78.50) + + +	69.00 (62.50–81.00) + +	67.00 (63.00–73.00)
Intervention group	59.00 (48.00–70.50)	55.00 (48.00–82.00)	41.60 (39.00–42.00)**	34.00 (32.00–36.25)***	41.00 (39.00–42.50)***	51.00 (45.00–56.50)
**MAP (mmHg)**						
Control group	80.00 (71.00–80.50)	74.00 (73.00–79.00)	79.00 (75.00–80.50) +	85.00 (74.00–88.00) + + +	80.00 (74.00–91.50) + + +	77.00 (74.50–82.50)
Intervention group	69.00 (63.00–87.00)	65.00 (61.50–91.50)	56.00 (51.00–57.00)**	48.00 (43.00–49.50)***	51.00 (49.50–53.00)***	67.00 (65.00–69.50)
**SV (ml)**						
Control group	49.00 (39.50–50.00)	46.00 (39.50–49.50)	40.00 (38.00–41.00)	47.00 (41.50–47.50)	45.00 (43.00–47.50)	45.00 (44.00–50.00) +
Intervention group	54.00 (48.00–56.00)	48.00 (41.00–54.50)*	52.00 (47.00–55.00)	55.00 (41.50–58.00)	51.00 (40.00–55.00)	32.00 (30.50–46.50)***
**CO (L/min)**						
Control group	4.75 (4.01–5.25)	4.44 (4.05–4.45)	4.13 (3.61–4.53)	4.62 (4.35–4.91)	5.27 (4.17–5.43)	5.50 (4.51–5.60)
Intervention group	5.09 (4.08–5.87)	4.78 (3.64–5.36)	4.80 (4.12–5.53)	5.86 (4.27–6.62)	5.68 (4.89–6.73)	6.01 (3.92–7.73)
**SVR (dyn s cm**^**-5**^**)**						
Control group	1297.00 (1190.00–1318.00)	1215.00 (1205.50–1284.50)	1362.00 (1118.50–1611.50)	1123.00 (986.50–1323.50)	1109.00 (1031.50–1470.00)	916.00 (902.00–1296.00)
Intervention group	826.50 (703.00–1258.00)	1196.00 (1167.00–1334.00)	757.00 (689.00–775.00)	522.00 (473.50–585.00)**	605.50 (567.00–580.00)*	830.00 (553.50–1098.00)
**SVV (%)**						
Control group	8.00 (8.00–11.00)	9.00 (8.00–12.00)	11.00 (9.00–11.50)	8.00 (7.00–11.50)	8.00 (6.50–9.00)	7.00 (6.00–9.50)
Intervention group	10.00 (9.00–13.00)	13.00 (11.00–13.50)	14.00 (13.00–15.00)	16.00 (14.00–17.00)	19.00 (16.00–26.50)**	20.00 (16.50–22.00)*
**PPV (%)**						
Control group	10.00 (8.50–14.50)	9.00 (9.00–12.50)	7.00 (7.00–11.00)	9.00 (7.00–13.50)	9.00 (6.50–11.00)	7.00 (6.50–9.50)
Intervention group	11.00 (8.00–15.00)	12.00 (10.50–17.50)	12.00 (11.00–17.00)	23.50 (18.00–26.50)**	19.50 (12.00–23.00)*	21.00 (19.00–24.00)*
**MSFP (mmHg)**						
Control group	16.88 (14.69–16.96)	16.90 (16.76–19.70)	18.84 (17.37–20.77)	16.92 (16.45–17.65)	19.33 (18.90–19.37)	18.95 (17.18–19.03)
Intervention group	16.03 (15.52–18.38)	16.86 (14.45–16.96)	14.94 (14.88–16.51)	15.42 (13.16–16.40)	17.37 (17.03–18.31)	14.99 (13.16–16.67)

Data are shown as median and interquartile range.

*CO* cardiac output, *DAP* diastolic arterial pressure, *HR* heart rate, *MAP* mean arterial pressure, *MSFP* mean systemic filling pressure, *PPV* pulse pressure variation, *SAP* systolic arterial pressure, *SVR* systemic vascular resistance, *SV* stroke volume.

T0, baseline in both groups; T1, 1 h after T0; T2, 2 h after T0; TH0, when an MAP < 50 mm Hg was reached. In the control group, this time was set at 3 h of observation. TH1, 1 h after TH0; TH2, 2 h after TH0.

+  = *p* < 0.05, +  +  = *p* < 0.01, +  +  +  = *p* < 0.001 to comparison between Control and Intervention Group. * = *p* < 0.05, ** = *p* < 0.01, *** = *p* < 0.001 to comparison between each time and the basal time on intervention group.

### PPV operative performance in each fluid challenge

The PPV was 11.0% (8.00–15.00%) at T0 and increased to 23.5% at TH0 (18.0–26.5%) in the endotoxin group (Table 1, Fig. 2A).

A decrease in the PPV operative performance as a predictor of fluid responsiveness was observed, as the AUC decreased from 0.95 at T0 to 0.60 at TH0. Moreover, its cutoff increased from 10.5 at T0 to 22.0% at TH0 (see Table 2). When noradrenaline infusion was initiated, the PPV operative performance increased to 0.75 at TH2.

**Table 2:** Operative performance of PPV in each fluid responsiveness.

Fluid challenge	Sensibility (%)	Specificity (%)	Threshold	AUC (95% CI)	Fluid responders (%)	*p*-value
T0	100	80	10.5	0.95 (0.81–1.00)	28.6	0.25
T1	100	100	10.5	1.00 (1.00–1.00)	28.6	0.25
T2	NA	NA	NA	NA	0	0
TH0	100	60	22	0.60 (0.17–1.00)	28.6	0.25
TH1	100	83	23	0.70 (0.26–1.00)	14.3	0.05
TH2	100	60	22	0.75 (0.35–1.00)	28.6	0.25

*AUC*, area under curve.

T0, baseline in both groups; T1, 1 h after T0; T2, 2 h after T0; TH0, when an MAP < 50 mm Hg was reached. In the control group, this time was set at 3 h of observation. TH1, 1 h after TH0; TH2, 2 h after TH0. *p*-value represents the difference between fluid responders and non-fluid responders.

### Association between PPV and other hemodynamic variables

The PPV model showed heteroscedasticity; therefore, it was necessary to transform PPV to the logarithm of PPV. The logarithm of PPV, which was obtained using a linear mixed model, showed a positive relationship with the baseline PPV; it was also higher in the endotoxin group. PPV showed an inverse relationship with SV, SVR, MAP, and MSFP. The analyses of all variables were statistically significant (*p* < 0,0001) (Additional file 1, Table S1). The AIC was 111.85, the BIC was 139.12, and the REML was -46.92. Additionally, the model showed homoscedasticity and normality of errors; therefore, its use was appropriate (Additional file 2, Figure S1).

The logarithm of PPV used in the present study was calculated as follows:

$$ \begin{aligned} {\text{Log}}\,{\text{PPV}} = & 4.26 + 1.73 \times 10^{ - 2} \left( {{\text{baseline}}\,{\text{PPV}}} \right) + 0.39\left( {{\text{endotoxin}}\,{\text{group}}} \right) {-}1.78 \times 10^{ - 2} \left( {{\text{SV}}\left( {{\text{ml}}} \right)} \right){-}7.46 \, \times 10^{ - 3} \left( {{\text{MAP}}\left( {{\text{mm}}\,{\text{Hg}}} \right)} \right) \\ & {-}2.05 \times 10^{ - 2} ({\text{MSFP}}\left( {{\text{mm}}\,{\text{Hg}}} \right) - 3.38 \times 10^{ - 4} \left( {{\text{SVR}}\left( {{\text{dyn}}\,{\text{s}}\,{\text{cm}}^{ - 5} } \right)} \right) \\ \end{aligned} $$

## Discussion

This study observed a decrease in the PPV operative performance and an increase in the cutoff as a predictor in the endotoxin group when an MAP < 50 mmHg was achieved. Moreover, when noradrenaline infusion was initiated, the PPV operative performance improved. These changes could be related to the inverse association between PPV, and SVR and MAP.

These findings should be considered when a fluid challenge will be performed in critically ill patients with hypotensive states, and PPV will be used as a predictor of fluid responsiveness. Some studies have reported several limitations of PPV as a predictor of fluid responsiveness^7,8^; however, we did not find studies that reported changes in the PPV operative performance in critically ill patients with low arterial pressure. We suggest that these findings can be explained by the relationship among MSFP, SVR, stressed volume, and unstressed volume. MSFP is one of the determinants of venous return, and this is determined by the relationship between the stressed/unstressed volume (30/70%)^19^ and SVR. In clinical conditions when SVR is low, MSFP decreases^20^, and the relationship between the stressed/unstressed volume changes, leading to a clinical condition of relative hypovolemia. PPV will be affected by MSFP; a decrease in MSFP would decrease the driving pressure for venous return, leading to a decrease in preload and SV. This clinical condition may not show a fluid response despite a high PPV value because the fluid load leads to an increase in the unstressed volume preventing an increase in MCFP, and hence an increase in venous return and SV^21^. Therefore, a high PPV does not mean that there is a high likelihood of fluid response in this setting. Moreover, when MAP increased due to noradrenaline infusion, the operative performance improved, hence confirming the relationship between the PPV operative performance and SVR described above.

We believe that these changes do not happen with stroke volume variation (SVV); since SVV is related to ventricular elastance^9^. Meanwhile, PPV is related to load arterial variables. Therefore, SVV should be a better predictor of fluid challenge in this setting. Since SVV is a central parameter and PPV is a peripheric parameter. Indeed, their PPV/SVV ratio (dynamic arterial elastance) could describe the relationship between the ventricular system and arterial system (ventricular-arterial coupling)^9^.

Our findings also showed an increase in the cutoff when an MAP < 50 mmHg was reached. Several cutoffs have been reported in the literature in different clinical settings; however, we are not aware of studies that reported an increase in the cutoff in patients with low arterial pressure. This parameter should be taken into account since it increases the amount of fluid used for reanimation, increasing the risk of fluid overload.

Other findings from our study included an inverse relationship between PPV and load arterial variables, such as SVR and MAP. Some studies support this relationship. Monge et al. showed an association between PPV and effective arterial elastance (Ea)^9^, a variable that summarizes the features of arterial vascular load in humans^10^. Other studies described a decrease in PPV after alpha-agonist infusion in experimental models of hemorrhagic shock, which could suggest a relationship between PPV and load arterial variables^22,23^. We suggest that this relationship could explain the low PPV operative performance when low arterial pressure was achieved.

Finally, we suggest that these findings should not be considered a limitation of PPV; instead, PPV should be considered a variable of preload dependency or a variable that allows us to recognize changes in the relationship of the stressed/unstressed volume, and the absence of changes related to a fluid challenge should alert us of the need for increased SVR. Thus, the risk of fluid overload will decrease.

The present study had some limitations. First, the evolution of PPV observed in our study may have been caused by increased vascular permeability, resulting in a decreased stressed blood volume, or by splenic red cell sequestration, as has been widely reported^24–27^. Second, we used the MSFP formula proposed by Parkin and Leaning^13^ instead of using an invasive measure of this variable. Nevertheless, this formula has yielded good results over time in swine models^26,28^. Moreover, the MSFP values found in the present study are similar to those reported in studies conducted in humans^29^. Third, our findings should not be extrapolated to all clinical settings since our model of endotoxin shock was characterized by low SVR with a compensatory increase in CO, such as early septic shock. Finally, Ea was not included in our regression model since this is calculated as MAP/SV or PP/SV; and, could have collinearity among Ea and MAP or SV. New studies are needed that allow the determination of the causes of the worsening of the PPV operative performance in this setting, assessing stressed/unstressed volume, MSFP, and venous return.

In conclusion, the PPV operative performance decreased, and the cutoff increased when MAP was < 50 mmHg. We also found an inverse association between PPV and load arterial variables, such MAP and SVR, that could be related to changes in the PPV operative performance.

## Supplementary Information

Supplementary Information 1.

Supplementary Information 2.
