# Supplementary material for: Changes of operative performance of pulse pressure variation as a predictor of fluid responsiveness in endotoxin shock
Source: Sci Rep. 2022 Feb 16;12:2590. doi: 10.1038/s41598-022-06488-x (PMC8850593; doi:10.1038/s41598-022-06488-x)
Supplement: Supplementary file 1 — Supplementary Information 1. [file 41598_2022_6488_MOESM1_ESM.docx]

Changes of operative performance of pulse pressure variation as a predictor of fluid responsiveness in a swine model of endotoxin shock

Jorge I Alvarado Sánchez, MD, MSc

Juan D Caicedo Ruiz, MD, MSc

Juan J Diaztagle Fernández, MD, MSc

Gustavo A Ospina Tascon, MD, PhD

Manuel I Monge Garcia, MD

Guillermo A Ruiz Narvaez, MV, MSc

Luis E Cruz Martínez, MD, MSc

| **Variable** | **Slope (sd)** | **Value t** | **Value p** |
| --- | --- | --- | --- |
| Intercept | 4.26(0.27) | 19.60 | P<0.0001 |
| PPV baseline (%) | 1.73.10^-2^(4.24.10^-3^) | 4.08 | P<0.0001 |
| Endotoxin Group | 0.39(0.05) | 6.93 | P<0.0001 |
| SV (ml) | -1.78.10^-2^(2.30.10^-3^) | -7.75 | P<0.0001 |
| MAP (mmHg) | -7.46.10^-3^(1.74.10^-3^) | -4.29 | P<0.0001 |
| MFSP (mmHg) | -2.05.10^-2^(7.03.10^-3^) | -2.92 | P<0.001 |
| RVS (dyn.s.cm-5) | -3.38.10^-4^(8.35.10^-5^) | -4.04 | P<0.0001 |

Table S1. The linear mixed effects model of the logarithmic of pulse pressure variation. MAP mean arterial pressure; MSFP, mean systemic filling pressure; PPV, pressure pulse variation; SVR, systemic vascular resistance; SV, stroke volume.
