# Supplementary figures and images for: Changes of operative performance of pulse pressure variation as a predictor of fluid responsiveness in endotoxin shock
Source: Sci Rep. 2022 Feb 16;12:2590. doi: 10.1038/s41598-022-06488-x (PMC8850593; doi:10.1038/s41598-022-06488-x)

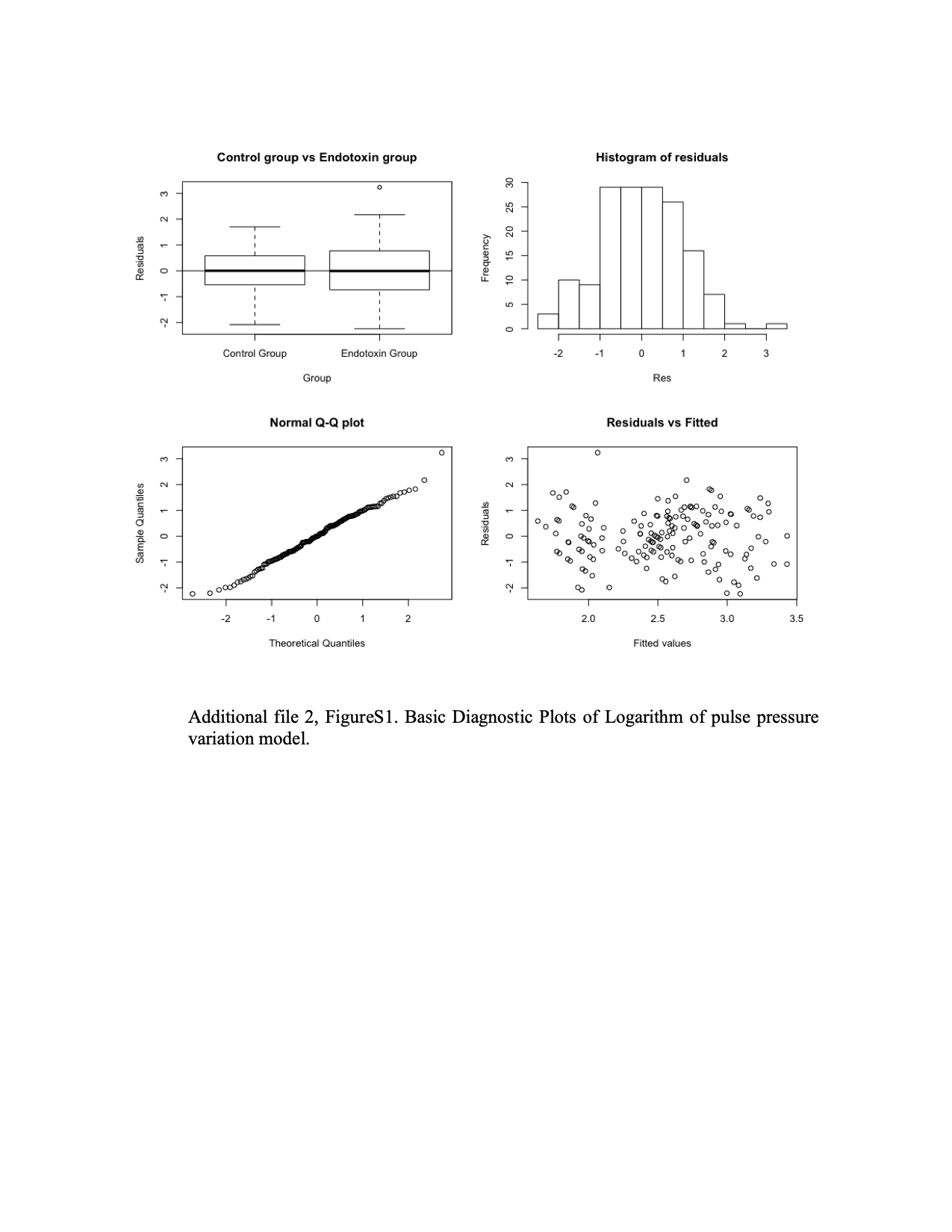

Supplement: Supplementary file 2 — Supplementary Information 2. [file 41598_2022_6488_MOESM2_ESM.tiff]
